# Investigating the Electric Field Lysis of Exosomes Immobilized on the Screen-Printed Electrode and Electrochemical Sensing of the Lysed-Exosome-Derived Protein

**DOI:** 10.3390/bios13030323

**Published:** 2023-02-27

**Authors:** Krishna Thej Pammi Guru, Nusrat Praween, Palash Kumar Basu

**Affiliations:** Department of Avionics, Indian Institute of Space Science and Technology, Thiruvanathapuram 695547, India

**Keywords:** electric field lysis of exosomes, immunoaffinity-based exosomes separation by screen-printed electrodes, electrochemical impedance spectroscopy, sensing of HER2, HSP70

## Abstract

It is important to isolate exosomes (<150 nm) from biofluid for diagnosis or prognosis purposes, followed by sensing of exosomal proteins. In the present work, exosomes are isolated from human serum by immobilizing on a Screen-Printed Electrode (SPE) followed by electric field lysis and electrochemical impedance spectroscopy (EIS)-based sensing of relevant exosomal proteins (HSP70 and HER2). Upon immobilization of exosomes on the surface, the role of different electrical signals (sinusoidal and square wave) in the lysis of exosomes was studied by varying the frequency and voltage. HSP70 was used for EIS to determine the optimal voltage and frequency for lysing the exosomes. It was observed that the low frequencies and, specifically, sinusoidal signals are ideal for lysing exosomes as compared to square signals. The relative quantity of HSP70 obtained by lysing with different voltages (sinusoidal waveform) was compared using Western blotting. After electric field lysis of the exosome with an optimized signal, HER2, a breast cancer biomarker, was detected successfully from serum by EIS. In the proposed technique, 3.5 × 10^8^ exosomes/mL were isolated from serum. With the limit of detection of 10 pg, the designed cell showed a linear detection of HER2 from 0.1 ng to 1 µg. It was observed from the results that the electric field lysis of exosomes not only plays a significant role in releasing the cargo protein but also improves the sensing of surface proteins associated with exosomes.

## 1. Introduction

Exosomes are nanovesicles (20–150 nm) secreted by cells and are considered significant in intercellular communication [1,2,3,4]. Exosomes contain a wide range of proteins, DNA, RNA, and miRNA [5] and they play a crucial role in signaling pathways that induce further exosome secretion by the destination cell [6]. As they are secreted from both healthy and tumor cells [7], they also play a crucial role in the prognosis and diagnosis of different diseases. They proved crucial in several cancers, including breast, pancreatic, lung, liver, colorectal, gastric, renal, bladder, ovarian, and prostate [8]. Besides cancer, exosomes also play a significant role in several diseases related to the heart, brain, immune system, Alzheimer’s, etc. [9,10]. However, for the diagnosis and prognosis process, it is important to separate exosomes from biofluid to perform sensing of exosome-associated proteins (cargo, surface, and transmembrane protein) for cancers or related diseases.

Using exosomes as biomarkers to improve patient care has been limited due to their small size and the extensive sample preparation time during isolation. Therefore, considerable research is in progress in developing methods for isolating exosomes from body fluids. The most widely used techniques to separate exosomes from biofluids are ultracentrifugation (UC), density gradient ultracentrifugation (DG-UC), and size-exclusion chromatography (SEC). Similarly, there are few commercially available kits for the isolation of exosomes (examples: ultrafiltration and nanoparticle-mediated separation). All these techniques need expensive instruments and expertise in using them. There are a few other methods such as magnetic separation, microfluidics, and immunoaffinity-based separation. The method of separation and the advantages and disadvantages of these techniques are discussed in our previous work published earlier [11]. Besides these techniques, researchers are exploring new methods that isolate and quantify exosomes in the same platforms [12]. Surface Plasmon Resonance techniques that rely on the phenomenon of total internal reflection aid in the identification of antigen and antibody interactions. Similarly, a nanoplasmonic exosome isolation platform based on collinear light transmission can target the marker proteins expressed on the surface of exosomes for isolation. Understanding the shift in spectra prior to and after exosome immobilization allows for the quantification of exosomes. Recently, waveguide structures were used for detecting the presence of extracellular vesicles (EVs) [13]. To immobilize the EV, polymer-coated waveguide structures were functionalized with suitable biomolecules, such as Biotin-Avidin. The shift in the wavelength of resonance can be used to confirm EV isolation. In addition to exosome quantification by light detection techniques, efforts are currently being made to isolate exosomes by surface markers and quantify them using various labelled or label-free electrochemical techniques [14]. One such technique is direct isolation of exosomes by Differential Pulse Voltammetry (DPV), in which anti-HER2 Antibody-immobilized electrodes were used for isolation and quantification of exosomes from cell culture media by DPV [15]. These techniques may not be effective if the surface of the exosomes lacks or is deficient in the expression of cancer-related marker proteins. Therefore, the lysis of exosomes, followed by the detection and quantification of their proteins, is significant in and of itself.

Traditionally, exosomes are lysed using a buffer (SDS, Triton X 100, Tween 20, and deoxycholate) [16]. However, it frequently results in a distortion of associated biomarkers. In addition, chemical-buffer-induced lysis may not be suitable for the microfluidic platform, because it requires a separate channel and chamber for lysis. Consequently, electric field lysis of exosomes may play a significant role in the release of exosome-related biomarkers without the need for additional channels and chambers. Recent research [17] utilized a microchannel with an integrated electrode array to lyse cells by applying a 20 V Peak-to-Peak signal at 1 MHz. However, based on our search, there are either no or very few reports on the electric field lysis of exosomes. Once the exosomes are lysed, their protein must be quantified using sensing techniques.

Existing literature compares various electrochemical techniques for detecting biological molecules [18,19,20]. Voltammetry [21], Differential Pulse Voltammetry [22], and Cyclic Voltammetry [23] are examples of such techniques. The majority of these methods employ an enzyme label, such as Alkaline phosphatase (AP), and a compatible substrate. AP operates on phosphate substrates such as phenyl phosphate (PP), 1-naphthyl phosphate (NP), and 3-indoxyl phosphate (3-IP). During the enzymatic conversion of the substrate, the byproducts create a passivation layer on the electrode, which reduces the sensing precision. HQDP is an alternative substrate that can be used with AP to prevent electrode surface passivation [24]. Electrochemical impedance spectroscopy can completely obviate the requirement for enzyme–substrate reaction when used for sensing (EIS).

The EIS-based label-free detection is attracting much interest for detecting enzymes, proteins, DNA, RNA, etc. [25]. EIS is especially useful for accurately estimating low protein concentrations. This makes the technique more applicable and practical when working with small sample volumes [26,27]. In EIS, the cell under test is perturbed with a small voltage and the corresponding current is measured. The ratio of the current measured and the voltage applied gives the impedance. The acquired data can be plotted using the Bode plot (magnitude vs. frequency and phase vs. frequency) and Nyquist plot (Z_R_ Vs Z_I_), represented by the combination of resistance and capacitance. The standard circuit used for this purpose is named the Randles circuit containing solution resistance (Rs), charge transfer resistance (Rct), and double layer capacitance (Csurf). The binding event can be detected by detecting the change in either the Rct or Csurf. In the absence of redox reactions (faradaic systems), Rct is very large and, hence, can be ignored [28]. In such systems, Csurf can be considered, which is a series combination of a constant-phase element (CPE) and double layer capacitance (Cdl) [29]. The CPE is due to surface modifications of the electrode, and its behavior is closer to that of the capacitor. It is well known that even the best circuit model cannot fit the EIS data exactly, and the same data can be represented in several different circuits [30,31,32,33]. Few studies have employed EIS techniques to detect the HER2 protein in serum [34] and saliva [33]. However, the detected HER2 is not derived by lysing the exosomes.

In this study, we utilized screen-printed Au working electrodes, which must be functionalized with an appropriate Antibody for exosome conjugation/separation (CD63) prior to lysing and exosome-derived protein conjugation (HSP70 and HER2) for sensing. To conjugate the Antibody to the SPE, thiolated PEG is commonly used to form self-adhesion monolayers [35] on gold. PEG in biological experiments has two benefits. The strong bond between the thiol group’s sulfur atom and gold surface is comparable to a covalent bond [36], making it suitable for multiple-wash processes. PEG interacts with proteins only after activation with EDC NHS; otherwise, it repels proteins from the surface. Incubation time and PEG layer thickness are directly proportional [37]. For PEGs under 5000 Daltons, protein repulsion is independent of spacer chain length if the chain density is 0.5 chains/nm^2^ [38].

As exosomes are charged particles just like cells, we followed a systemic approach to lyse the exosomes by an electric field (sine and square waves at different frequencies) and detected the lysed protein (HER2 and HSP 70).

Based on our investigation, it has been determined that there is no detailed study of electric-field-induced exosome lysis followed by EIS-based detection of the lysed protein. In this study, a detailed optimization of the electric field lysis of the immobilized-exosome is presented at different voltages and frequencies (sinusoidal/square wave) on the SPE followed by EIS-based label-free detection of HER2 and HSP 70.

## 2. Materials and Methods

**Figure 1:**
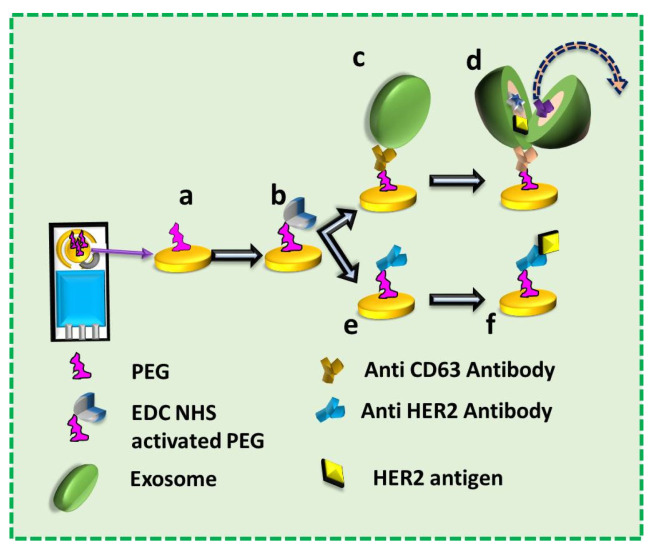
The flow of the work. a: Polyethylene Glycol (PEG) functionalized on the Screen-Printed Electrode (SPE); b: Activation of COOH group of PEG with EDC NHS; c: SPE functionalized with anti-CD63 Antibody and immobilized with exosome; d: Lysing of exosomes on SPE by electric field; e: SPE functionalized with anti-HER2 Antibody; f: Immobilization of exosomal protein (HER2) on the SPE functionalized with anti-HER2 Antibody.

In the present research, we investigated the isolation of exosomes on the SPE (SPE) followed by lysing of the exosomes immobilized on the surface by applying the electric field and label-free sensing of the exosomal protein on the SPE. The steps of the work are as follows: PEG immobilization on the SPE’s working electrode, activation of the COOH of the PEG by EDC NHS chemistry, conjugation with anti-CD63 Antibody, incubation of serum for immobilization of exosomes on the SPE, lysis of exosomes by a sinusoidal/square wave signal, and sensing of the HER2 and HSP70 proteins obtained after lysing of exosomes using another SPE. A detailed flow chart is provided in Figure 1.

### 2.1. Materials

SPEs were procured from Metrohm (DRP-250BT). 2-(*N*-morpholino) ethanesulfonic acid MES), *N*-(3-Dimethylaminopropyl)-*N*-ethylcarbodiimide (EDC), Poly (ethylene glycol) 2-mercaptoethanol ether acetic acid (PEG 3500 Da), *N*-Hydroxysuccinimide (NHS), bovine serum albumin, Halt Protease, and phosphatase inhibitor were procured from Sigma Aldrich(St. Louis, MO, USA). Amersham ECL reagent was obtained from GE Health Care(Chicago, IL, USA). Clot activator tubes and powdered Non-fat milk were procured from local vendors. All other consumables and reagents were reagent grade.

Primary Antibodies: Anti-CD63 (Cat No. PAB25155) was obtained from Abnova for Western blotting. Anti-CD63 (Cat No. BS-1523R) (for functionalizing the SPEs), Anti-HSP70 (Cat No. Mab 33-3800), and Anti-TSG101 (Cat No. PA5-31260) were procured from Thermo Fisher(Waltham, MA, USA). Anti-HER2 (Cat No. SAB4500785) and HER2 Antigen (Cat No. H3040) were procured from Sigma. Anti-CD63 (Cat No. AB134045) and Anti-HER2 (Cat No. Ab16901) (for Western blotting) were procured from Abcam.

Secondary antibodies: Anti-rabbit (Cat No. ab131366) and anti-mouse (Cat No. ab131368) HRP were obtained from Abcam(Cambridge, UK).

### 2.2. Methods

#### 2.2.1. Immobilizing PEG on the SPE

SPEs were washed in DI water. PEG solution of different concentrations (28.5 µM, 285 µM, 857 µM, 1.42 mM) was prepared in DI water to optimize the concentrations and 200 µL was used for incubation on the SPE for 2 h at room temperature. After incubation, the excess PEG was removed by washing the surface with DI water.

#### 2.2.2. Functionalization of Antibody on the SPE

The SPE was incubated with 200 µL of PEG solution for 2 h at room temperature followed by rinsing with Deionized water. The COOH group of the PEG was activated using EDC NHS chemistry. In 200 µL of MES buffer of pH 6.17, 20 µL of 10% NHS and 14 mM of EDC were mixed and incubated for 30 min at room temperature, followed by incubating the wafer with 200 µL of SPB (Sodium Phosphate Buffer) containing 1 µL of Antibody for 30 min at 4 °C. After the incubation, the SPE was washed with washing buffer for 10 min at 4 °C to remove excess antibodies and replaced immediately with SPB. Different experiments were performed by varying the concentrations of EDC to 28 mM, 56 mM, 141 mM, and 282 mM for optimizing the concentrations.

#### 2.2.3. Immobilization of Exosomes on the SPE for Isolating the Exosome from Serum

An amount of 100 µL of serum was diluted with 100 µL of SPB and used for incubation on the SPE, which was functionalized with anti-CD63 Antibody. The incubation was performed at different incubation times (10 min, 20 min, and 30 min) at 4 °C to optimize the process, followed by washing the SPE with washing solution for 10 min at 4 °C. Finally, the electrode surface was replaced with SPB.

#### 2.2.4. Exosome Lysis and Electrochemical Impedance Spectroscopy (EIS)

After immobilizing the exosomes on the SPE, the surface was washed with washing solution for 10 min at 4 °C. The solution was replaced with SPB mixed with protease and phosphatase inhibitor to a final concentration of 1×. To optimize the lysis process, square and sine waves of different frequencies (10 Hz, 500 Hz, 1 KHz) and with different amplitudes (50 mV, 500 mV, 2 V) were applied to the SPE for 5 min at room temperature. Next, the exosome lysate solution was used for incubation with the SPE, which was already functionalized with anti-HSP 70 Antibody. After incubation, the SPE was washed with washing solution for 10 min at 4 °C and replaced with 200 µL of SPB. Electrochemical Impedance Spectroscopy (EIS) was performed with the SPE using a sinusoidal waveform of amplitude 10 mV with frequencies ranging from 10^5^ Hz to 1 Hz. In addition to these experiments, further lysis experiments were performed with a sinusoidal waveform at 10 Hz with voltages of 50 mV, 500 mV, 750 mV, and 1 V. All the lysate solutions were used for Western blotting and probed for HSP70.

#### 2.2.5. Generation of Standard Quantification Curve with HER2

EIS experiments were performed with known concentrations of HER2 to generate a standard curve in order to use it for comparison with unknown protein concentrations in serum collected from a donor. Here, a SPE functionalized with Anti-HER2 Antibody was incubated with different concentrations (1 µg, 0.1 µg, 0.01 µg, 1 ng, 0.1 ng, 10 pg) of HER2 dissolved in 200 µL of SPB for 30 min at 4 °C. The incubation was followed by washing the SPE with washing solution for 10 min at 4 °C and immediately replacing the washing solution with SPB. After the washing, the SPE was used for EIS using a sinusoidal waveform of amplitude 10 mV with frequencies ranging from 1 Hz to 10^5^ Hz. The EIS reading from all the experiments was used to generate an equivalent circuit. The CPE values obtained from the circuits corresponding to different concentrations of HER2 were used to generate a mathematical expression relating the concentration of HER2 (x in µg) by measuring the value of the CPE (CPE = 6.54(x) + 6.29).

#### 2.2.6. Serum Preparation

All the serum was prepared from blood collected from volunteers. The blood was allowed to clot in clot activator tubes, in an upright position, for 1 h at room temperature. This clot activator tube was then spun at 7000× g for 20 min to separate serum and cells. After collecting the serum from the tube, 100 µL aliquots were prepared and stored at −20 °C. Before performing the experiment, 100 µL of SPB was added to 100 µL of serum to reduce the density of the serum.

#### 2.2.7. Elution of Exosomes from the Surface of the IDE for NTA Quantification

To quantify the number of exosomes immobilized on the SPE surface, a SPE functionalized with 1 µL of anti-CD63 Antibody was incubated with a 200 µL solution containing 100 µL of serum and 100 µL of SPB for 30 min at 4 °C. After incubation, the SPE was washed with washing solution for 10 min at 4 °C. To elute the exosomes immobilized on the surface, the SPE was incubated with 40 µL of 0.1 M glycine buffer of pH 3 for 50 min at room temperature. After incubation, the solution was immediately mixed with 1 µL of 1 M NaOH to neutralize the pH of the solution. The eluted exosomes were used for NTA analysis using NanoSight NS300, Malvern Panalytical Instruments(Malvern, UK).

#### 2.2.8. Western Blotting

The lysis was performed in 50 µL of SPB upon immobilization of exosomes on the SPE surface. Five different lysis experiments were performed with the sinusoidal waveform of frequency 10 Hz with voltages of 50 mV, 500 mV, 750 mV, and 1 V. SDS sample loading buffer was mixed with the exosome-lysed solution and heated in a block heater at 97 °C for 10 min. In addition, 10% SDS-PAGE was used to run the samples, which was followed by blotting in PVDF membrane. At room temperature, the blot was blocked for 1 h, with 5% non-fat milk. Suitable primary Antibody was used to probe the PVDF membrane overnight at 4 °C, which was followed incubation with HRP-conjugated secondary Antibody. ECL reagents were used to develop the blots.

## 3. Results

In the present work, the SPE was functionalized with PEG containing SH and COOH groups at its terminals. The COOH group of the PEG was activated using EDC NHS chemistry to make it suitable for conjugation with Antibodies (CD63, HSP70, HER2). The exosomes were immobilized on the surface by incubating the serum on the SPE functionalized with the anti-CD63 Antibody. Several experiments were performed for lysing the exosomes by applying sinusoidal and square waves of different voltages and frequencies, as mentioned in the Section 2.2. After each lysis experiment, Electrochemical Impedance Spectroscopy (EIS) was performed for the exosomal protein HSP70, and the corresponding data were fit using a circuit.

### 3.1. Optimization of Washing Solution to Prevent Non-Specific Adsorption

As EIS is extremely sensitive to minute protein concentrations, it is crucial to prevent the nonspecific binding of proteins from serum on the SPE during exosome immobilization. A wide range of experiments were performed to study and eliminate the non-specific adsorption of antibodies from the gold surface. The presence of Antibody on the gold surface can be confirmed calorimetrically using TMB-HRP chemistry, which involves the change in the color of the substrate from colorless to blue. Hence, secondary Antibody tagged with HRP was used for finding the appropriate washing solution.

Detailed optimization experiments were carried out with different washing solutions to wash the surface, which included: SPB (0.25 mM, 0.1 M, 0.5 M, and 1 M), NaCl (0.5 M and 1 M), 0.1% Tween 20, Triton X-100 (0.1%, 1%, and 10%), and solution with a combination of 5% Tween 20 and 5% Triton X-100.

A bare gold surface was incubated with 1 µL of secondary Antibody for 30 min followed by washing with 0.25 mM SPB for 15 min. After incubating TMB with the washed SPE, the TMB changes from colorless to blue. As the change in the color of TMB indicates the presence of Secondary Antibody on the SPE surface, the experiments were repeated by increasing the number of washing steps to two and three times, with different durations (15 min, 30 min, and 45 min). Similar experiments were performed by using different salt and detergent solutions (SPB, NaCl, Tween 20, Triton X-100). After washing the SPEs and incubating them with TMB, a significant color change was noticed in TMB solution, which indicates more adsorption of Antibody on the Au surface of IDE. Finally, it was concluded that removal of the adsorbed proteins from the gold surface, even with highly concentrated salts or detergents, is a difficult task and, hence, in order to avoid the non-specific adsorption, PEG was immobilized on the surface of the SPE.

### 3.2. PEGylation of SPE

There are several ways to functionalize gold to immobilize proteins and other biomolecules. Each affects sensor sensitivity and storage capacity. Direct adsorption of Protein A onto a gold surface is one technique, followed by conjugation of an antigen-specific antibody. Proteins are amphiphilic and readily adsorb to gold through hydrophobic and electrostatic interactions. Protein A cannot bind to goat IgG, rat IgG, or mouse IgG, so Protein G is used [39]. Direct adsorption of proteins may cause a random orientation, reducing antigen binding sites and antibody binding [40]. Protein A or G can be replaced with biotin to adsorb on gold. Biotin/avidin or biotin/streptavidin interactions are stable [41], similar to covalent bonds, and unaffected by temperature, pH, or detergents. As biotin, avidin, and streptavidin are temperature-sensitive and must be kept at a certain temperature [21], we opted to functionalize the Au surface of the SPE with Poly Ethylene Glycol (PEG).

PEG solutions of different concentrations (28.5 µM, 285 µM, 857 µM, 1.42 mM) were prepared in 5 mL of DI water. The bare gold surface of the SPE was incubated with PEG solution for 2 h and rinsed with 25 mM SPB. The SPE was then incubated with SPB mixed with 1 µL of secondary Antibody for 30 min. After incubation, the SPE was washed with 0.1% Triton X-100 solution prepared in 25 mM SPB for 10 min. Following washing, the SPE was incubated with TMB solution. Experiments were also performed by changing the washing solutions (0.1% Tween 20, 0.5 M NaCl, 0.1 M SPB). After washing, TMB solution was incubated with the SPE. Altering the washing solutions was also the subject of experiments (0.1% Tween 20, 0.5 M NaCl, 0.1 M SPB). The color change of TMB solution was compared to the color change of the SPE, which was directly adsorbed with secondary Antibody. The results demonstrated that SPEs functionalized with PEG alters the color of TMB more slowly than those without PEG. This suggests that the formation of a PEG coating on the SPE can reduce the non-specific adsorption of proteins onto the SPE.

PEG concentrations of 285 µM, 857 µM, and 1.42 mM produce comparable visible results, and it was concluded from the above experiments that 0.1% Tween 20 and 0.1% Triton X-100 are more effective than 0.5 M NaCl and 0.1 M SPB at removing the adsorbed secondary Antibody. As Tween 20 is a gentler detergent than Triton X-100, 0.1% Tween 20 was used to remove nonspecific adsorption. We found that 2 h of incubation is sufficient for the formation of the PEG layer on the gold surface, despite the fact that other studies used 48 h.

### 3.3. Optimization of EDC Concentrations for Activating the COOH of PEG

EDC NHS coupling chemistry is well known and is best carried out at lower pH values. Hence, MES buffer with pH 6.17 was chosen for our work as it can maintain acidic pH [11]. While keeping the NHS concentration fixed, a detailed optimization was carried out by varying the EDC (14 mM, 28 mM, 56 mM, 141 mM, 282 mM) followed by washing cycles and immobilization of 1 µL of secondary Antibody tagged with HRP. After introducing TMB to the SPE, a significant color change was noticed only at 28 mM EDC, indicating the presence of secondary Antibody on the SPE. Increasing the concentration of EDC increases the pH level of the solution, thereby reducing the half-life of the intermediate compound significantly less, making it unsuitable for interaction with the Antibody. A low concentration of EDC might not be sufficient to activate the COOH of the PEG. Hence, a 28 mM concentration of EDC was used for conjugation of Antibody to the SPE in all further experiments.

**Figure 2:**
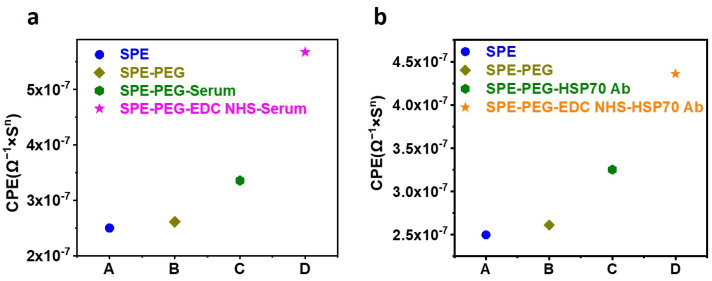
SPE functionalized with PEG and incubated with serum and Antibody. (**a**) CPE of PEG-functionalized SPE, incubated with serum before and after EDC NHS activation. The figure shows a significant increase in the binding of serum protein to the SPE after EDC NHS activation. (**b**) CPE of PEG-functionalized SPE, incubated with primary Antibody before and after EDC NHS activation. The change in the CPE confirms the conjugation of primary Antibody to the SPE after EDC NHS activation.

In addition to TMB HRP-based detection, the SPE functionalized with 285 µM PEG was utilized for EIS sensing to confirm the activation of COOH of the PEG with EDC NHS. The COOH of the PEG was activated with a 28 mM concentration of EDC, which was then incubated with serum. Figure 2 depicts the equivalent CPE obtained from the corresponding Nyquist plots following EIS sensing. In Figure 2a, the EIS results reveal that the conjugation of the serum protein to the EDC-activated PEG on the SPE significantly increases the CPE. As a standard, the CPE obtained from the SPE without EDC activation was utilized. Figure 2b demonstrates the binding of HSP70 Antibody to EDC-activated PEG. The results indicate two conclusions: first, PEG increases the sensitivity of the SPE; second, EDC NHS activation of PEG is required for protein conjugation.

### 3.4. Saturation of SPE with Anti-CD63 Antibody for Maximizing the Exosomal Immobilization

**Figure 3:**
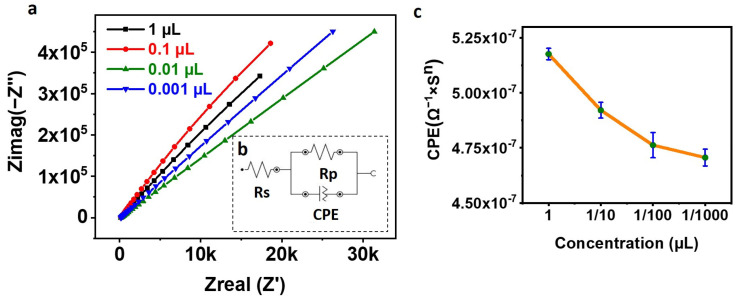
(**a**) Nyquist Plot. The figure shows the Nyquist plot of SPE functionalized with different concentrations of anti-CD63 Antibody. (**b**) Randles Circuit used for fitting the Electrochemical Impedance Spectroscopy (EIS) data. (**c**) Plot of CPE against the concentration of the CD63 Antibody. The figure shows the reduction in the net capacitance, i.e., CPE with the reduction in the concentration of the Antibody used for conjugating the Screen-Printed electrode (SPE).

A saturation test was performed to maximize the amount of CD63 Antibody on the SPE, which, in turn, increases the number of exosomes that can be captured on the SPE surface. After activating the COOH of the PEG with EDC NHS, the SPEs were incubated with different concentrations (1 µL, 1/10 µL, 1/100 µL, and 1/1000 µL) of anti-CD63 Antibody for 30 min, followed by washing for 10 min and tested for EIS. Figure 3a shows the Nyquist plots, Figure 3b shows the circuit used for fitting the Nyquist plots, and Figure 3c shows the plot of CPE vs. concentration. From Figure 3a, it can be observed that increasing the Antibody concentration reduces the net impedance as observed by other researchers also [25]. Figure 3c shows a linear relation between the CPE and the amount of anti-CD63 Antibody used for functionalizing the SPE. Increasing the concentration of the Antibody to the PEG increases the net capacitance, thereby reducing the net impedance. We chosen to functionalize the SPE with 1 µL of CD63 Antibody to maximize the exosome capture.

**Figure 4:**
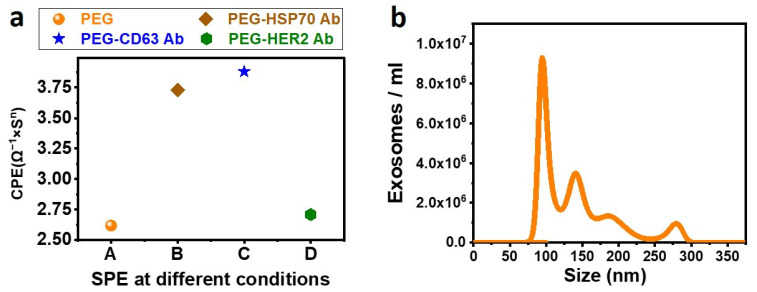
(**a**) CPE is obtained from an equivalent Randles Circuit at different conditions of the Screen-Printed Electrode (SPE). SPE functionalized with PEG, COOH of the PEG activated with EDC-NHS, and SPE conjugated with different Antibodies (anti-HSP70, anti-CD63, and anti-HER2) are fitted using a Randles circuit, and the corresponding CPE is plotted. (**b**) NTA characterization of exosomes. The results show isolation of 3.5 × 10^8^ exosomes/mL serum. The maximum number of particles is found to be at 100 nm.

Figure 4a presents the SPE under different conditions such as PEG before EDC-NHS activation and the CPE obtained after conjugating the SPE with different types of Antibodies. It can be seen that the CPE value increased after conjugating the antibodies on the functionality surface, which agrees with all our agreements.

To confirm the incubation time for immobilizing the exosomes on the SPE, 1 µL of anti-CD63 Antibody was incubated with human serum for 30 min at 4 °C followed by Western blot, and a band was noticed. Therefore, 30 min of incubation time is sufficient for immobilizing the exosomes on the SPE. The exosomes immobilized on the SPE were eluted and quantified using NTA, and it was observed that 3.5 × 10^8^ exosomes are isolated from 100 µL of human serum with a mean diameter of 148 nm. The maximum number of particles is approximately 100 nm in size. The corresponding data are shown in Figure 4b.

### 3.5. Exosomal Lysis with Sine and Square Waves at Different Voltages and Frequencies

**Figure 5:**
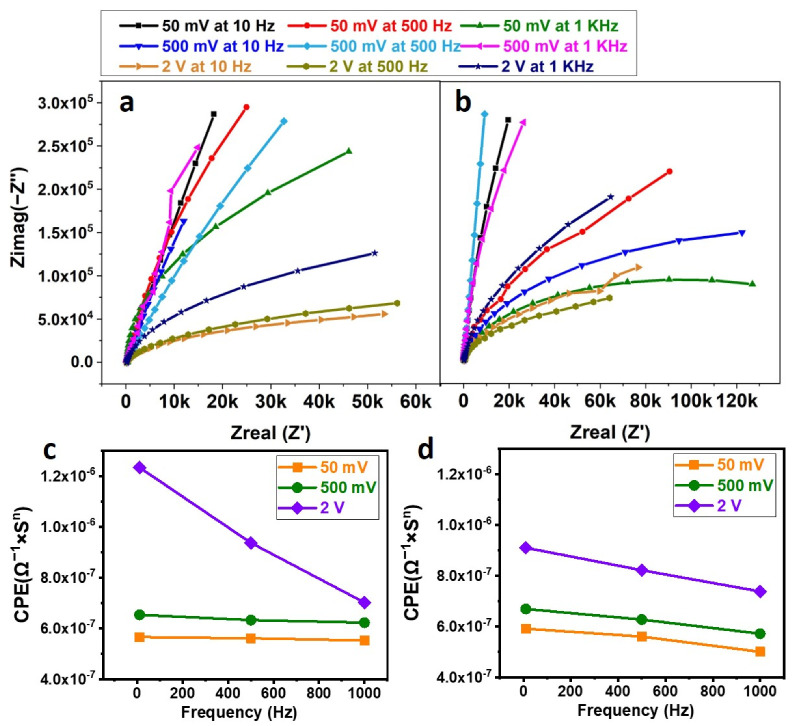
(**a**) Nyquist Plot Corresponding to Sine wave Lysis; (**b**) Nyquist Plot Corresponding to Square Wave Lysis; (**c**) Plot of CPE vs. frequency at different voltages of Sine wave; (**d**) Plot of CPE vs. frequency at different voltages of Square wave. The exosomes immobilized on the Screen-Printed Electrode (SPE) are lysed using sine and square waves of different voltages and frequencies. The lysate solution is incubated with anti-HSP70-immobilized SPE, and Electrochemical Impedance Spectroscopy (EIS) is performed. The EIS data are fit in a Randles circuit and the corresponding capacitor (CPE) values obtained are plotted against the frequency.

After incubating the exosome on the SPE, detailed experiments were performed to lyse the exosomes with different voltages (50 mV, 500 mV, and 2 V) and frequencies (10 Hz, 500 Hz, and 1 KHz). The exosome lysate solution was incubated with the SPE functionalized with anti-HSP70 protein for 30 min at 4 °C, and finally, after washing, the buffer was replaced with SPB, and EIS readings were taken. Figure 5a,b correspond to the Nyquist plots, for sine and square waves, which were fit using the Randles circuit, and the corresponding CPEs were plotted against the frequency in Figure 5c,d. The values are summarized in Table 1.

After applying lysis voltage to the exosome, conjugated on the SPE, it should become lysed and the contents released into the buffer solution (lysate). After collecting and incubating the lysate solution with the SPE functionalized with Anti-HSP70 Antibody, a change in the CPE indicates antigen binding to the Antibody. As the antigen layer acts as another capacitor layer in series with the existing capacitance, a higher CPE indicates a greater amount of antigen bound to the SPE. From Figure 5c,d, it can be seen that, for any particular voltage, the CPE value corresponding to a frequency of 10 Hz is higher compared to those of 500 Hz and 1 KHz (irrespective of sine or square wave). The results indicate the presence of more antigens conjugated to the SPE for 10 Hz frequency compared to other higher frequencies. This can be due to the fact that the channels within the exosomal membrane are being influenced only if they are exposed to the same voltage for a longer duration.

Similarly, for a 10 Hz signal, it can be seen that increasing the magnitude of the lysing voltage increases the CPE, indicating more antigen binding to the SPE. Increasing the applied voltage to the SPE increases the electric field within the solution. The surface of exosomes, which is charged in nature, might become perturbed more for higher voltages. Hence, higher voltages could be more useful in lysing the exosomes. From Figure 5c,d, it can be observed that the sinusoidal waveform has more CPE compared to that of the square wave. The gradual reduction in the voltage (caused by sine wave) plays a better role in exosomal lysing than sudden changes in the voltage (caused by square wave). Hence, it was assumed that the sine wave is better in lysing the exosomes than the square wave.

### 3.6. Western Blotting and EIS of Exosomal Lysate Solutions of Sine Wave

**Figure 6:**
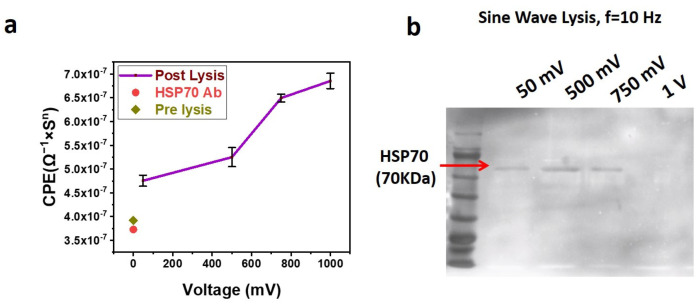
(**a**) Sine Wave Lysis of exosomes at 10 Hz Frequency with different voltages. The exosomes immobilized on the Screen-Printed Electrode (SPE) are lysed using sine waves of different voltages. The lysate solution is incubated with anti-HSP70-immobilized SPE, and Electrochemical Impedance Spectroscopy (EIS) is performed. The EIS data are fit in a Randles circuit, and the corresponding capacitor (CPE) values obtained are plotted against the frequency. (**b**) Western blot performed with samples obtained after Lysing exosomes using the sinusoidal waveform of 10 Hz frequency. The blot shows the Western results of SDS-PAGE loaded with samples in which exosomes are lysed with different voltages of 50 mV, 500 mV, 750 mV, and 1 V. After immobilization of exosomes on the SPE, 50 µL of SPB is used to lyse for 5 min. A 50 µL solution is used for Western blotting.

To study the role of exosome lysis in detail, further experiments were performed with a sine wave at a frequency of 10 Hz with different voltages (50 mV, 500 mV, 750 mV, and 1 V). After applying a lysing voltage to the exosomes, immobilized on the SPE, the lysate solution was used for EIS and Western blotting. For EIS, the lysate solution was directly incubated with the SPE functionalized with anti-HSP70 Antibody for 30 min at 4 °C, followed by washing and sensing. The corresponding EIS results are shown in Figure 6a. The increasing CPE value with an increase in the lysis voltage indicates better lysis of exosomes at higher voltages.

For Western blotting, the lysate solution was treated with a sample loading buffer for electrophoresis. After electrophoresis, the blot was probed for HSP 70, which is shown in Figure 6b. The blot shows the presence of HSP 70 only for lysing voltages of 50 mV, 500 mV, and 750 mV but not for 1 V. We assume that the voltages of 1 V and beyond are too high, resulting in the complete rupture of exosomes and significant deformation to its proteins, resulting in fragments that are not detectable by primary antibodies. Hence, it can be assumed that voltages in the range of 50 mV to 500 mV are optimum for lysing the exosomes. Though 500 mV and 750 mV lysate solutions give significant bands for HSP 70, it can be seen from Figure 6b that the band intensity for HSP70 is maximum for exosomes lysed with 500 mV. HSP 70 is an interior protein for exosomes bound to the surface. As exosomes are immobilized on the SPE via an Antibody, one side of the exosome will be closer to the SPE surface. The impact of the voltage applied to the SPE will be relatively high in the regions of the exosome closer to the SPE surface and continues reducing as the distance from the SPE increases. Hence, it can be assumed that the HSP 70 proteins that are bound to the exosomal surface, which is closer to the SPE surface, receive more impact due to the applied voltage, hence becoming fragments and undetectable by Western blotting.

On the other hand, a band exists for HSP 70 (Figure 6b) for 50 mV also. As the exosomal membrane is charged in nature, a mild voltage such as 50 mV might have partially opened the channels on the membrane, leading some of its protein to the external solution. Hence, we assumed that voltages ranging from 50 mV to 500 mV are suitable for lysing the exosomes.

**Figure 7:**
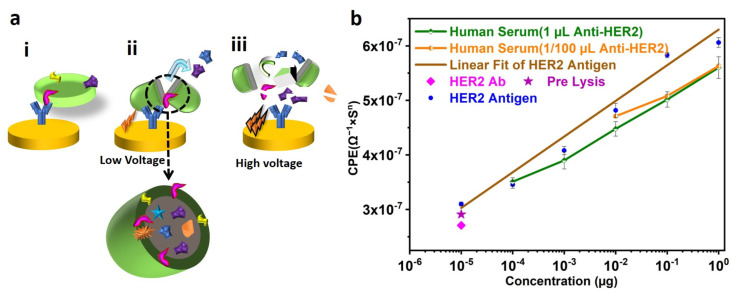
(**a**) Mechanism of exosome lysis by Electric Field. (**i**) Exosome immobilized on the SPE functionalized with anti-CD63 Antibody. (**ii**) Low-Voltage Lysis of Exosome. Due to the applied voltage on the SPE, the charged nature of exosomes becomes partially disturbed, resulting in the release of its contents. (**iii**) Rupture of exosomes due to High Voltage. Exposing the solution containing exosomes to high voltages can rupture the exosomes as well as their proteins to fragments, making them unusable for further processing. (**b**) Generation of HER2 Quantification curve and quantification of unknown concentration of HER2. A known concentration of HER2 antigen is immobilized on the SPE, functionalized with anti-HER2 Antibody. The corresponding data obtained after the Electrochemical Impedance Spectroscopy (EIS) sensing are used to fit the Randles circuit. The capacitance (CPE) from the circuit is plotted against the concentration of HER2 (CPE = 6.54 (µg of HER2) + 6.29)). Serum from the volunteer is used for isolating exosomes and Lysing. The lysate solution is used for (EIS) sensing and compared with the standard curve. Approximately similar results are seen for SPEs conjugated with saturated (1 µL) and non-saturated (1/100 µL) concentrations of Anti-HER2 Antibody.

Hence, it can be concluded that electric field lysing can be used as a replacement for detergent lysis for extracting the exosomal protein for analysis purposes. The schematic of the lysis process due to the electric field is presented in Figure 7a(i) for better understanding.

Although some literature quantifies the HER2 protein, we have been unable to locate any that quantifies HER2 in lysed exosomes. EIS can detect HER2 concentrations between 5 and 50 pg/mL in saliva [33]. This technique would be more useful if salivary HER2 estimation standards were also established for various stages of cancer, similar to serum HER2 levels (19 ng/mL [42]). The advantages and disadvantages of various techniques that detect HER2 are tabulated in Table 2.

### 3.7. Quantifying HER2 Protein from Human Serum by Isolating, Electric-Field Lysing, and Sensing

HER2 is a protein that exists in three domains: Extracellular Domains, transmembrane region, and tyrosine kinase domain [43]. Hence, Anti-HER2 could be used to immobilize the exosomes. However, CD63 is one of the most abundant transmembrane proteins on exosomes, which we found to be a better choice for maximizing exosome immobilization on the surface. To understand the role of lysing on the exosome-surface-protein (HER2), similar experiments were performed but without using lysates. In this case, SPB (as there is no lysate) from the exosome-immobilized SPE was used for incubating the SPE, conjugated with an anti-HER2 Antibody. EIS sensing was performed and CPE values were extracted from the equivalent impedance modeled as mentioned earlier. Figure 7a(ii) shows a negligible difference between the CPE values, confirming minimal nonspecific binding to the anti-HER2 Antibody. However, there is a significant increase in CPE after using electric-field-induced lysate, which indicates proper conjugation of Antibody and HER2. This may be due to the fact that electric field lysis generates multiple fragments of HER2 from the exosomes and, hence, HER2 contents in the lysate increased, resulting in more conjugation of HER2 and sensing. Therefore, it can be concluded that even though the HER2 is a surface protein, lysing is required to enhance the sensitivity of the sensors.

To quantify the HER2 protein from the serum, the known concentrations (i.e., 1 µg, 0.1 µg, 0.01 µg, 1 ng, 0.1 ng, 10 pg) of HER 2 were used for immobilization on the anti-HER2-conjugated SPE. The concentration of HER2 and the corresponding CPE values are plotted in Figure 7a(ii). The CPE of each of these concentrations of HER2 was used to model a mathematical expression to relate unknown HER2 concentrations (CPE = 6.54(x) + 6.29). From Figure 7a(ii), a linear relationship between the concentration of HER2 and the CPE can be seen (Figure S1 in the Supplementary Material depicts the equivalent bode plot for various HER2 concentrations).

In order to check the amount of HER2 from a random individual, blood was collected, and serum was used for isolating and lysing exosomes. The serum was used to immobilize the SPE’s exosomes, followed by lysis using a sinusoidal wave at 500 mV in 50 µL of SPB. Different concentrations, i.e., 1 µL, 0.1 µL, 0.01 µL, and 0.001 µL, of the exosome lysate were used for quantification of HER2 using the SPE immobilized with an anti-HER2 Antibody. The corresponding data are shown in Figure 7a(ii). It was observed that the serum contains HER2 of 0.6 µg and it is linear in nature. The advantages and disadvantages of various HER2 protein detection techniques are presented in Table 2. According to our knowledge, no literature quantifies HER2 extracted from lysed exosomes. Figure 7a(ii) demonstrates comparable results for SPEs functionalized with 1 µL and 1/100 µL dilutions of HER2 Antibody, thereby reducing the amount of Antibody needed and making the technique more sensitive and cost-effective. As the sensor developed in this study can detect HER2 concentrations between 1 µg and 10 pg, it is suitable for studying HER2 in breast cancer patients during diagnosis and treatment.

## 4. Conclusions

Exosomes are known to play a crucial role in cancer progression as messengers. Isolating exosomes and analyzing their contents contribute to a greater understanding of cancer. This study analyzed the lysis of exosomes with sinusoidal and square waveforms at different frequencies and voltages in great detail. The results demonstrated that the low-frequency sinusoidal waveform is superior to the square waveform for lysing exosomes. Using electrochemical impedance spectroscopy, the exosomal protein obtained after the lysis of exosomes was detected and represented as an equivalent circuit. Moreover, it was observed that electric field lysis is not only necessary for releasing the cargo protein, but also advantageous for enhancing the sensing device’s sensitivity. Using a standard curve derived from known HER2 protein, the protein from the lysed exosome was quantified. The proposed technique isolated 3.5 × 10^8^ exosomes/mL of serum. As the designed system showed a linear detection of HER2 from 0.1 ng to 1 µg, it can be used for detecting HER2 in cancer patients for real-time prognosis and diagnosis.

We believe the conducted experiments demonstrate the interaction between exosomes and electrical signals. As this technique for lysing exosomes uses a defined voltage and frequency, we believe that its precision, accuracy, and repeatability will be superior to those of detergent-based lysing methods. Further experiments can be conducted to investigate the role of various signal types, at a wider frequency range, and by varying the duration of the lysis. To make the entire procedure more compact, a platform that supports exosome isolation, lysing, and sensing can be developed. As a label-free technique, the proposed method can be easily integrated with microfluidics techniques not only for lysing exosomes, but also for sensing.

## Figures and Tables

**Table 1:** Capacitance Values (CPE) were obtained from the circuit after incubating the exosome lysate solution on the SPE functionalized with anti-HSP70 Antibody.

Voltage	Sine Wave			Square Wave		
	10 Hz	500 Hz	1 KHz	10 Hz	500 Hz	1 KHz
50 mV	5.92 × 10^−7^	5.60 × 10^−7^	5.00 × 10^−7^	5.65 × 10^−7^	5.60 × 10^−7^	5.52 × 10^−7^
500 mV	6.69 × 10^−7^	6.27 × 10^−7^	5.72 × 10^−7^	6.53 × 10^−7^	6.32 × 10^−7^	6.22 × 10^−7^
2 V	9.10 × 10^−7^	8.22 × 10^−7^	5.48 × 10^−7^	1.23 × 10^−6^	9.36 × 10^−7^	7.01 × 10^−7^

**Table 2:** Advantages and Disadvantages of Various Existing Techniques for detecting HER2 protein.

Technique	Advantages	Disadvantages	Limit/Range of Detection/Source
Differential Pulse Voltammetry(DPV) [15]	Direct Exosome isolation and sensing on SPE	Labeled Technique, Accurate quantity of HER2 may not be detectable	4.5 × 10^5^ exosomes. Range: not mentioned. Medium
Aptamer-based Non-Faradaic EIS [34]	Label-free.	Aptamers are not easily available compared to Antibodies.	0.2–2 ng/mL; Serum
Sandwich immunoassay using Linear Sweep Voltammetry [43]	Well-known process steps and can be accurate. In this method, HER2 is detected without isolation of exosomes by utilizing HER2 fragments of serum.	Enzyme label is required. Needs two Antibodies for detection. More processing steps.	15–100 ng/mL; Serum.
Electrochemical Impedance Spectroscopy [33]	Does not need blood for detection. Label-free.	Serum levels for HER2 should be greater than 19 ng/mL [43] for breast cancer patients.	5-40 pg/mL; Saliva
Our Technique	Label-Free. HER2 is specific to exosomes. Can be extendable to specifically isolate all types of Extracellular Vesicles, by varying the Antibody on the SPE for immobilization.	A second SPE should be used for detecting the protein derived from exosome lysate.	Linear range: 0.1 ng to 1 µg. Limit of detection: 10 pg. Serum

## Data Availability

Not applicable.

## Supplementary Materials

The following supporting information can be downloaded at: https://www.mdpi.com/article/10.3390/bios13030323/s1, Figure S1. Bode plot of known concentration of HER2. A known concentration of HER2 antigen is immobilized on the SPE, functionalized with anti-HER2 Antibody. After Electrochemical Impedance Spectroscopy(EIS) sensing, the corresponding magnitude and phase of the bode plot data were plotted against frequency.
