# Supplementary material for: Investigating the Electric Field Lysis of Exosomes Immobilized on the Screen-Printed Electrode and Electrochemical Sensing of the Lysed-Exosome-Derived Protein
Source: Biosensors (Basel). 2023 Feb 27;13(3):323. doi: 10.3390/bios13030323 (PMC10046613; doi:10.3390/bios13030323)
Supplement: Supplementary file 1 [file biosensors-13-00323-s001.zip › biosensors-2079882-supplementary.pdf]

biosensors

Supplementary

Investigating the Electric Field Lysis of Exosomes Immobilized on the Screen-Printed Electrode and Electrochemical Sensing of the Lysed-Exosome-Derived Protein

Krishna Thej Pammi Guru, Nusrat Praween and Palash Kumar Basu *

Department of Avionics, Indian Institute of Space Science and Technology, Thiruvanathapuram 695547, India
* Correspondence: palashkumarbasu@iist.ac.in

1. Bode plot of known concentrations of HER2

Different concentrations (1 µg, 0.1 µg, 0.01 µg, 1 ng, 0.1 ng , 10 pg) of HER2 protein is functionalized on the SPE conjugated with anti HER2 Antibody. Subsequently, the SPE was employed for Electrochemical Impedance Sensing (EIS), and the corresponding Bode plot is shown in Figure S1. The unknown concentration of HER2 protein in the blood of volunteers could be estimated by comparing the obtained phase and frequency curves.

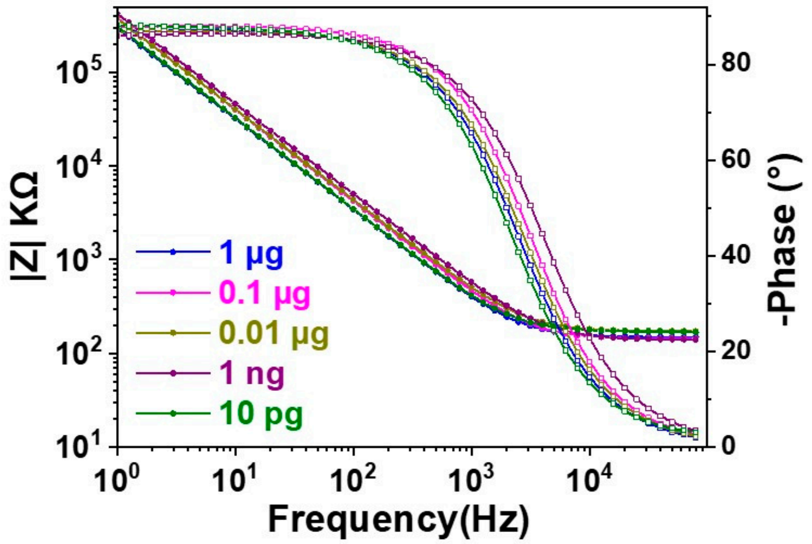

Figure S1. Bode plot of known concentration of HER2. A known concentration of HER2 antigen is immobilized on the SPE, functionalized with anti-HER2 Antibody. After Electrochemical Impedance Spectroscopy(EIS) sensing, the corresponding magnitude and phase of the bode plot data were plotted against frequency.
